# Cost-effectiveness of Antiviral Stockpiling and Near-Patient Testing for Potential Influenza Pandemic

**DOI:** 10.3201/eid1402.070478

**Published:** 2008-02

**Authors:** M. Ruby Siddiqui, W. John Edmunds

**Affiliations:** * Health Protection Agency, London, UK

**Keywords:** Pandemic, influenza, decision modeling, antivirals, point-of-care systems, cost effectiveness, QALY, research

## Abstract

A decision analytical model was developed to investigate the cost-effectiveness of stockpiling antiviral (AV) drugs for a potential influenza pandemic in the United Kingdom and the possible role of near-patient testing in conserving AV drug stocks. Under base-case assumptions (including a fixed stockpile that was smaller than the clinical attack rate), the treat-only option (treating all symptomatic patients with AV drugs) would be considered cost-effective (£1,900–£13,700 per quality-adjusted life year [QALY] gained, depending on the fatality scenario), compared with no intervention (nonintervention but management of cases as they arise). The test-treat option (testing all symptomatic patients but treating those with positive tests results only) would result in moderate gains in QALYs over the treat-only option but at relatively large additional costs. Stockpiling sufficient AV drugs (but not near-patient tests) to treat all patients with clinical cases would be cost-effective, provided AV drugs are effective at preventing deaths from pandemic influenza.

## Abstract 2

Storing sufficient antiviral drugs to treat all patients with clinical cases is cost-effective.

Many countries are ordering stockpiles of antiviral (AV) drugs for use in a potential influenza pandemic. The United Kingdom recently announced the procurement of 14.6 million courses of oseltamivir, enough for almost 25% of the population (*1*).

The timing of an influenza pandemic cannot be predicted (the most recent pandemics occurred in 1918, 1957, and 1968/69). Because AV drugs have a limited shelf-life, long-term maintenance of stockpiles may constitute a significant cost. Similarly, the size (clinical attack rate [CAR]) of a pandemic cannot be accurately foreseen. Therefore, triaging of patients with influenza-like illness (ILI) may be essential to conserve limited AV drug stocks. Possible triaging methods include near-patient testing (rapid diagnostic tests at the point of care). This study assessed the cost-effectiveness of stockpiling AV drugs for a potential influenza pandemic and, in the event of a pandemic, also assessed the use of near-patient testing in the management of AV drugs.

## Methods

**Figure 1:**
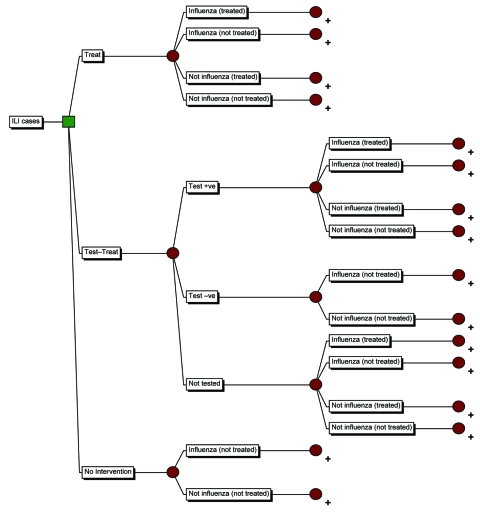
Decision analytical model tree of treatment strategies for patients with an influenza-like illness (ILI) during an influenza pandemic. All branches culminate in the subtree (indicated with +). QALY, quality-adjusted life year; CAR, clinical attack rate.

A decision analytical model (Figure 1) was constructed to compare the costs and quality-adjusted life year (QALY) loss associated with 3 potential strategies for the management of patients with ILI in the United Kingdom: 1) do not treat with AV drugs and manage complications if they arise (no intervention), 2) treat all patients with AV drugs (treat only), or 3) test then treat those who test positive for influenza with AV drugs (test-treat). Precision Tree (Palisade Corporation, Ithaca, NY, USA) running in Microsoft (Redmond, WA, USA) Excel was used to construct the model and @Risk (Palisade Corporation) was used to perform the probabilistic sensitivity analysis.

### Epidemiologic Scenarios

Baseline epidemiologic scenarios were based on the UK Department of Health Pandemic Contingency Plan (*2*), which assumes a cumulative CAR of 25% over 1 wave lasting 15 weeks. The demand on general practitioners (GPs) and accident and emergency departments (A&Es) would require an alternative means for AV drug distribution (*2*). Patients with ILI were therefore assumed to have received AV drug therapy by visiting teams or call-in centers. Of these, because of secondary complications, 5% were assumed to have consulted further with a GP and 5% with A&E departments. Also, among ILI patients, 0.55% were expected to be hospitalized. All model parameters are summarized in the Appendix Table.

Background ILI rates were deduced from the mean ILI consultation rates in England and Wales observed by GP-based sentinel surveillance from 1985–2003 (*4*) and the estimated proportion (28%) of clinical ILI case-patients who consulted with GPs in interpandemic periods (*5*). Epidemic influenza is seasonal with a higher incidence in winter (wk 40–12) than summer (wk 13–39). The mean weekly ILI incidence was assumed to have remained unchanged during the pandemic, resulting in a cumulative incidence of 3.1% (winter) or 1% (summer). An additional value of 4% for midwinter (wk 49–8) was also considered in the sensitivity analyses. It is possible that a pandemic strain would out-compete the existing epidemic influenza strains and the background ILI rate would fall. However, other pathogens (e.g., respiratory syncytial virus and rhinovirus) that contribute to ILI would remain unaffected (*22*). The background ILI rate was therefore assumed to remain unaltered. The base-case analysis assumed a winter pandemic, giving a probability (PrF) of 89% (CAR/(CAR + background ILI rate) that a case of ILI was pandemic influenza .

Two fatality scenarios were assumed, on the basis of the 1918 influenza pandemic (1918 scenario), which had an overall case-fatality ratio (CFR) of 2.3%, and the 1957 and 1968/69 influenza pandemics (1957/69 scenario) which had an average CFR of 0.3%, with most deaths occurring in the elderly (Appendix Table).

Three pandemics occurred in the 20th century, which suggests that a pandemic occurs approximately every 30 years but that this is a random (Poisson) process. For the base-case analysis, we assumed that the next pandemic would take place in 30 years; this figure was varied from 1 to 50 years in the sensitivity analyses.

### Antiviral Treatments

The neuraminidase inhibitor oseltamivir was selected as the AV treatment of choice because it is cheaper and easier to stockpile than zanamivir and has been shown to dominate zanamivir in cost-utility analyses (*23*). Cyclic amines (amantadine and rimantadine) were not considered because resistance to these drugs has emerged in influenza A virus (H5N1) with pandemic potential (*24*). The rate of adverse events after the use of neuraminidase inhibitors is low and therefore was not considered in this analysis (*12*).

Meta-analyses of oseltamivir efficacy studies were compared, and the 2003 study by Kaiser et al. was selected for the parameterization of this model because it included the greatest number of subjects (*5*,*15*,*20*). No data were available for AV efficacy in reducing influenza-related deaths so the same efficacy as that for reducing influenza-related hospitalizations was assumed. Assuming all GP and accident and emergency (A&E) consultations were attributable to the development of complications (*2*), the probability of complications and the probability of hospitalizations (given no AV therapy) were calculated by using the odds ratios from the oseltamivir meta-analyses (*15*). The probability of complications for noninfluenza ILI patients was considered the same as for untreated influenza patients, but the probability of hospitalization for noninfluenza ILI patients was considered to be 40% that of untreated influenza patients (*15*).

We assumed that for both the treat-only and test-treat strategies all qualifying patients were given AV drugs and were tested until the stockpile ran out. No epidemiologic differences were assumed between those who received AV drugs and those who did not because the stockpile was depleted. Neuraminidase inhibitors are only recommended for use within 48 hours of symptom onset (*25*). We assumed that the efficiency of AV drug distribution would be such that 70% of ILI patients would receive timely AV drugs; this varied from 30% to 97% in our sensitivity analyses (Table). Those receiving AV drugs after 48 hours were assumed to derive no benefit.

**Table:** Total NHS costs and QALY loss (discounted at 3.5%) resulting from an influenza pandemic occurring in 30 years assuming 1918 or 1957/69 CFR*

Treatment program	Pandemic influenza cases (millions)	Pandemic influenza deaths (millions)	Discounted NHS costs (million £)	Discounted QALY loss (millions)	Incremental cost per QALY (£)
1918 scenario					
No intervention	15	0.344	113	2.23	
Treat only	15	0.236	1,361	1.56	1,861†
Test-treat	15	0.231	2,356	1.53	31,031‡
1957–69 scenario					
No intervention	15	0.044	113	0.395	
Treat only	15	0.030	1,361	0.303	13,668†
Test-treat	15	0.030	2,356	0.299	227,896‡

*NHS, National Health Service; QALY, quality-adjusted life years; CFR, case-fatality ratio. †Cost per QALY gained over no intervention program. ‡Cost per QALY gained over treat-only program.

The shelf-life of oseltamivir is currently 5 years. However, tests are being conducted to increase this to 6 years (*6*). Shelf-life was varied in our sensitivity analyses between 4 and 6 years.

For base-case analyses, we assumed the AV drug stockpile to be 14.6 million courses (*1*), giving an 87% probability of case-patients receiving AV drugs (size of stockpile/total ILI cases). Hence, the size of the AV stockpile would be limited under the treat-only strategy.

### Near-Patient Tests

A number of near-patient influenza tests are currently available (*7*–*10*). To reflect the continual improvement in test technology, a composite test was constructed based on the best performance of currently available tests (89.5% sensitivity and 99.8% specificity) (Directigen Flu A+B, Becton Dickinson, Sparks, MD, USA) (7,*12*), a shelf-life of 2 years (Quickvue, Quidel Corporation, San Diego, CA,USA) (*9*), and a cost of £7 per test (Biostar Flu OIA, Inverness Medical-Biostar Inc, Louisville, CO, USA) (*8*). Parameter distributions for test sensitivity and specificity were deduced from a meta-analysis of near-patient tests (*12*), but distributions were assumed for the shelf-life and cost.

Although the United Kingdom has not accumulated a stockpile of near-patient tests, we assumed it to be the same size as the AV drug stockpile in the base-case analysis. The probability of being tested (test-treat option) was 87% (size of stockpile/total ILI cases).

### Costs

As recommended in the United Kingdom, costs were analyzed from the perspective of the healthcare provider, the National Heath Service (NHS). Future costs and benefits were discounted at the current rate of 3.5% per annum and all costs were in 2004 pounds sterling (£1 = ≈US$1.8) (*14*,*26*).

Mean unit costs per GP consultation**,** hospitalization, and A&E attendance for ILI were deduced from standard sources (*17*,*19*). We assumed that no additional costs were associated with death. The costs of complications leading to GP consultation or hospitalization were assumed to be the same for pandemic influenza and nonpandemic ILI.

The unit cost of an AV treatment course was assumed to be £16 (*16*) for the treat-only option and £16.87 (because of excess AV drugs) for the test-treat option (fixed stockpiles). The unit cost of a near-patient test was assumed to be £7 (*7*), and storage was £1 per unit (AV drug course or test) per year for both programs. Administration costs for the distribution of AV drugs or near-patient testing by visiting teams or call-in centers were assumed equivalent to the mean cost of a home visit by a district nurse, health visitor, health care assistant, or practice nurse (£15.75 per test or AV drug course) (*17*). Units were assumed to be procured >2 years (*1*) and replenished at expiration of shelf-life.

### Health Benefits

Health benefits were assessed by using QALYs. QALY loss associated with uncomplicated ILI were calculated study by O’Brien et al. (*20*) assuming a normal health score of 0.85 (Table). QALY loss associated with the development of complications was assumed to be the sum of QALY loss associated with uncomplicated ILI and that associated with pneumococcal pneumonia (outpatient) because this was the most likely complication (*21*). Similarly, QALY loss associated with hospitalization was considered the sum of QALY loss associated with uncomplicated ILI and that associated with pneumococcal pneumonia (inpatient) (*21*).

The mean discounted QALY loss associated with pandemic influenza death was estimated by using age-specific CFR under the 2 death scenarios and background life expectancy by age weighed by age-adjusted quality-of-life scores (Appendix Table) (*27*). QALY loss associated with noninfluenza death was assumed to be the same as that associated with *S. pneumoniae* death (a mean discounted QALY loss per noninfluenza death of 6.09 from 1980–2000) (*3*).

### Base-Case Assumptions

In the base-case analysis, the cost-effectiveness of the potential strategies was compared under the assumptions of fixed AV and test stockpiles (14.6 million units), a CAR of 25%, and a time to pandemic of 30 years. There is no threshold for cost-effectiveness in the United Kingdom, although the National Institute for Clinical Excellence will probably reject an intervention on cost-effectiveness grounds if the cost per QALY gained is in excess of £25,000–£35,000 (*28*). For ease of exposition, we used a simple threshold of £30,000 per QALY to define a cost-effective intervention.

## Results

### Base-Case Analysis (Fixed Stockpiles)

In our base-case model, we estimated that in a pandemic, ILI would develop in 28.1% of the population (16.8 million persons) (including 15 million pandemic influenza patients). Under the high CFR conditions of the 1918 scenario, ≈344,000 deaths would occur compared with ≈44,000 deaths under the 1957/69 scenario (Table). These scenarios would result in the loss of ≈2.2 or 0.4 million discounted QALYs, respectively, with a total discounted cost to the NHS of £113 million if no treatment program were initiated (no intervention). The treat-only program would reduce this loss by 700,000 or 90,000 QALYs at a cost of ≈£1,900 or £13,700 per QALY gained for the 1918 and 1957/69 scenarios, respectively, well below the £30,000 threshold. The test-treat program would further reduce this loss slightly by 30,000 or 4,000 QALYs but at a high cost of ≈£31,000 or £228,000 per QALY gained over the treat-only alternative. The test-treat option would be unlikely to be considered because cost-effectiveness is highly dependent on the fatality scenario.

### Univariate Sensitivity Analysis of the Treat-Only Program (Fixed Stockpile)

**Figure 2:**
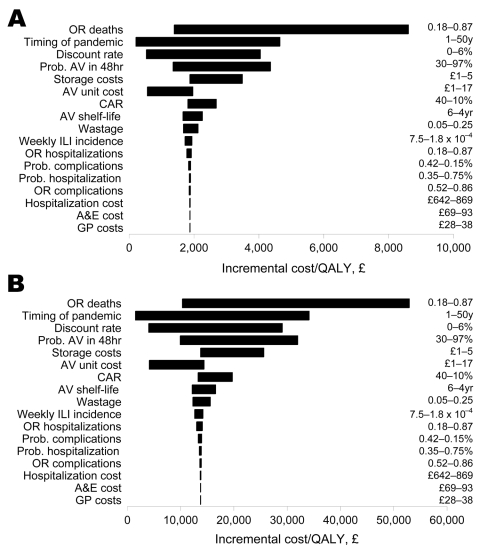
Univariate sensitivity analyses of the incremental cost-effectiveness of the treat only strategy over the no-intervention strategy to model parameters under the 1918 scenario (A) and 1957/69 scenario (B). OR, odds ratio; AV, antiviral; CAR, clinical attack rate; ILI, influenza-like illness; A&E, accident and emergency department; GP, general practitioner; QALY, quality-adjusted life year.

Because the treat-only program was the most cost-effective program under both fatality scenarios, we carried out a univariate sensitivity analysis of the incremental cost-effectiveness of this program to variability in model parameters (Appendix Table). AV drug efficacy for reducing complications and hospitalizations had minimal effect on the cost-effectiveness of the treat-only program, but this strategy was highly sensitive to AV drug efficacy for reducing death (Figure 2). This was due to the relatively high QALY loss associated with pandemic influenza death (94% and 69% of the total QALY loss for the 1918 and 1957/69 scenarios, respectively). Because the value of this parameter is unclear, further studies of the potential protective effect of AV drugs against death are essential.

The timing of the pandemic and the discount rate were influential parameters. However, variation in the timing of the epidemic is unlikely to change the recommendation that the treat-only strategy is cost-effective. If an epidemic occurs in 45 years, the costs per QALY gained would be approximately £3,800 and £28,000 for the 2 fatality scenarios (discount rate of 3.5%), still below the £30,000 threshold. At a discount rate of 6%, the treat-only option would be cost-effective for up to 30 years.

The efficiency of AV drug distribution is likely to be important. The program would however remain cost-effective if the probability of receiving AVs within 48 hours did not drop below 35% (£3,700 or £27,000 per QALY gained for the 1918 and 1957/69 scenarios), respectively.

The treat-only program was slightly more cost-effective in the summer than the winter or midwinter as the probability of pandemic influenza being the cause of ILI was higher (96%, 89%, and 84%, respectively). Wastage (wasted quantities, fraud, theft) of AV drug supplies as high as 25% had little effect on cost-effectiveness of the treat-only option (Figure 2).

A lower CAR (15%) reduced the cost-effectiveness of AV drugs because some of the stockpile would not be used (surplus). Increasing the CAR above 25% had no effect on programs with fixed stockpiles because the same number of deaths and complications would be prevented at the same cost (the proportion of deaths prevented would be reduced, but the absolute number would remain the same).

### Threshold Conditions for Test-Treat Option (Fixed Stockpiles)

**Figure 3:**
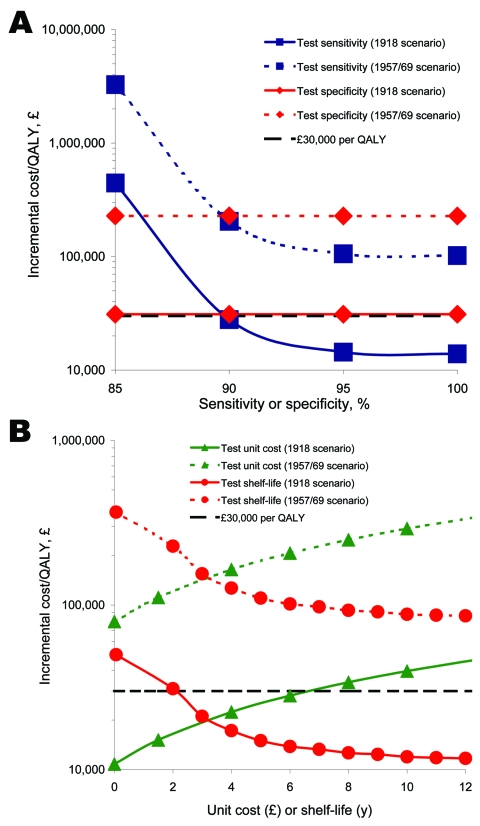
Univariate sensitivity analyses of the incremental cost-effectiveness of the test-treat strategy over the treat only strategy to A) near-test sensitivity and specificity and B) near-test unit cost and shelf-life. The test-treat program becomes cost-effective below the cost-effectiveness threshold (£30,000 per quality-adjusted life year [QALY] gained).

For high CARs, where a fixed AV drug stockpile is less than the expected demand (as in the base-case), near-patient tests could be used to better target therapeutic courses. A univariate sensitivity analysis of the incremental cost-effectiveness of test-treat over treat only to variability in the near-patient test parameters, test sensitivity, specificity, unit cost, and shelf-life, was carried out. Under the 1918 scenario the test-treat strategy would require test sensitivity to exceed ≈90% (Figure 3, panel **A**) and a test unit cost below £6 or a shelf-life above 3 years (Figure 3, panel **B**) to be considered cost-effective. Test specificity would have little effect on the incremental cost-effectiveness because it has no effect on QALY loss. Under the 1957/69 scenario test-treat would never cross the cost-effectiveness threshold even with a 100%-sensitive or 100%-specific test, a test cost as low as £0, or a shelf-life as high as 4 years.

### Probabilistic Sensitivity Analysis (Fixed Stockpiles)

**Figure 4:**
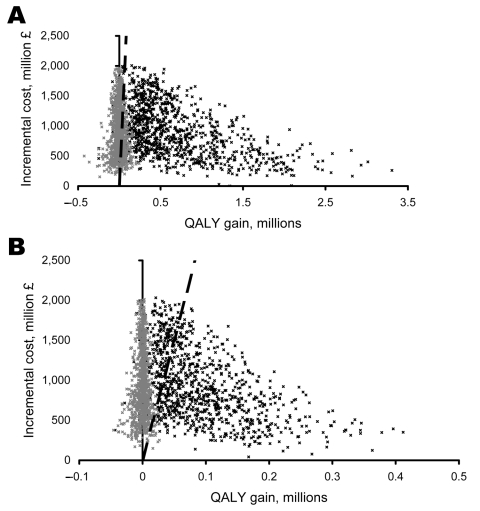
Probabilistic sensitivity analysis of the incremental cost-effectiveness of the treat-only over the no-intervention strategy and the test-treat strategy over the treat-only strategy for the A) 1918 and B) 1957/69 death scenarios (1,000 iterations). Cost-effective strategies lie to the right of the cost-effectiveness threshold (£30,000 per quality-adjusted life year [QALY] gained).

Model parameters were varied (Appendix Table) in a probabilistic sensitivity analysis, which suggests that for fixed AV drug and test stockpiles, the probability is high that the treat-only option would be cost-effective, irrespective of the fatality scenario (Figure 4). The test-treat option would result in small QALY gains (and often losses) but at substantial additional costs. The probability of this strategy being cost-effective is low compared with the treat-only option, particularly for the 1957/69 fatality scenario.

### Incremental Cost-effectiveness during a Pandemic Wave (Fixed Stockpiles)

The probability that an ILI case will be due to pandemic influenza will vary over the time course of a pandemic (assumed to peak between wk 6 and 7 for a wave lasting 15 wk) (*2*). Therefore, near-patient testing may be useful during early stages of a pandemic when clinical judgment is low and inappropriate AV administration is high. The cost-effectiveness of test-treat over a pandemic wave was analyzed.

**Figure 5:**
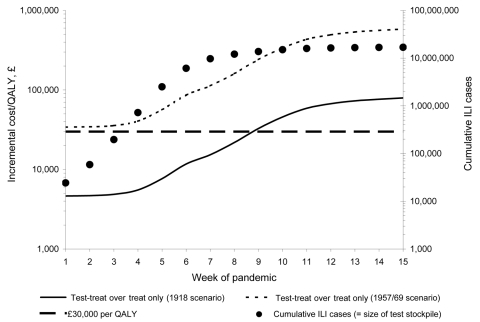
Incremental cost-effectiveness of the test-treat strategy over the treat-only strategy during a pandemic wave (antiviral [AV] stockpile = 14.6 million courses, test stockpile = number of cumulative influenza-like [ILI] cases, clinical attack rate = 25%). QALY, quality-adjusted life year.

The AV drug stockpile was assumed to remain fixed at 14.6 million courses (*1*), and the test stockpile was varied with the cumulative number of ILI cases expected per week of the pandemic wave. Figure 5 shows the total incremental cost-effectiveness of the test-treat strategy over treat only for each test stockpile for a CAR of 25%. Test-treat would be cost-effective (<£30,000 per QALY gained) for test stockpiles up to 12.1 million (the expected no. of cumulative ILI cases at wk 8 of a pandemic) under the 1918 scenario. Test-treat may even be considered for test stockpiles up to 13.7 million (wk 9 of a pandemic) as the cost-effectiveness was ≈£32,700 per QALY gained. However, under the 1957/69 scenario test-treat would not be cost-effective at any stage of the pandemic, although it may be considered for test stockpiles up to ≈35,000 (wk 2 of a pandemic) as the cost was ≈£34,000 per QALY gained.

For a CAR of 15% (data not shown), test-treat would not be cost-effective throughout a pandemic as the AV drug stockpile would exceed demand (cumulative ILI cases). For a CAR of 35% (data not shown), although test-treat would be cost-effective for test stockpiles up to 16.4 million (wk 8) under the 1918 scenario, it would not be cost-effective at any stage of the pandemic under the 1957/69 scenario. Therefore in the short-term, stockpiling enough tests for the first 2 weeks of a potential influenza pandemic (≈35,000 tests) could help conserve limited AV drug stockpiles. However, this is highly dependent on the CAR and CFR. In the long-term, a more cost-effective plan would increase AV drug stockpiles to cover expected demand (CAR + background ILI + wastage, see optimal Stockpiling).

### Optimal Stockpiling

**Figure 6:**
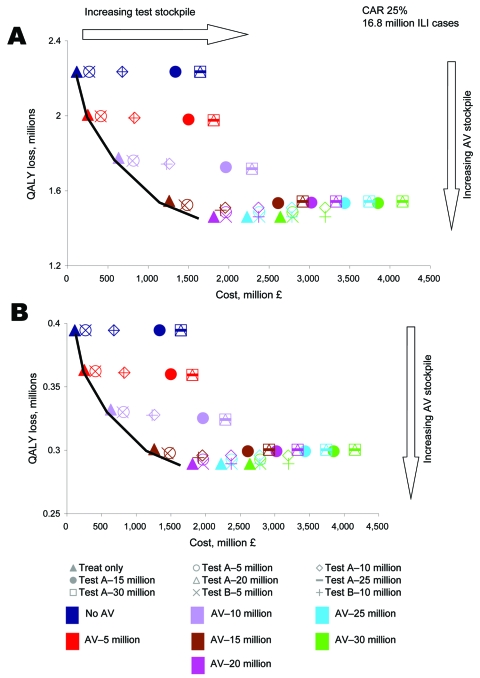
Optimal cost-effectiveness of antiviral (AV) and test stockpiling (0–30 million units) for a clinical attack rate (CAR) of 25% under the A) 1918 and B) 1957/69 scenarios. The composite test (Test A) and a perfect test of 100% sensitivity and 100% specificity (Test B) are included. The most cost-effective strategies lie on the efficiency line. ILI, influenza-like illness; QALY, quality-adjusted life year.

In the long term, it may be more cost-effective to increase stockpiles to cover expected demand (CAR + background ILI + wastage). Figure 6 and the Appendix Figure show the expected costs and QALY losses under a range of different AV drug and test stockpiles and CARs (base-case test characteristics and AV drug efficacy assumed). Each point represents 1 scenario (test and AV drug stockpile size). Points on the efficiency line were potentially cost-effective strategies. Strategies that increase cost but reduce QALY loss (moving from left to right on the efficiency line) should be considered until the slope of the line exceeds the threshold of £30,000 per QALY (efficiency line ends). For each CAR, this process suggested that the optimal strategy was treat only, stockpiling enough AV drugs to meet demand (CAR plus background ILI plus AV drug wastage). Therefore, for a CAR of 25%, the optimum stockpile was ≈20 million AV drugs only (Figure 6) because the expected number of ILI cases would be 16.8 million (15 million of which would be pandemic influenza) and the expected AV drug wastage would be 2.2 million. The test-treat strategies were never on the efficiency line, even for a perfect test (100% sensitivity and specificity), because they resulted in similar QALY loss as treat only but at increased costs. Indeed, when the size of the AV drug stockpile exceeded the demand, test-treat resulted in increased QALY loss (if the test is not 100% sensitive), because some true pandemic influenza case-patients would be denied treatment even though there was a surplus of AV drug courses.

## Discussion

This study demonstrates that stockpiling AV drugs for a treat-only program is likely to be a cost-effective strategy in preparation for a potential influenza pandemic, even if the pandemic occurs many years from now, assuming that AV drugs provide some protection against death. However, under current UK planning assumptions (CAR 25%), the AV stockpile would be too small (at 14.6 million courses) to treat all cases of ILI. Near-patient testing is unlikely to be a cost-effective approach to conserving AV stocks but might be considered early in a pandemic. A more cost-effective strategy would be to increase the stockpile of AV drugs. Since CARs in excess of 30% have been observed in pandemics (*29*), increasing the stockpile to cover this possibility may be both prudent and cost-effective. Indeed, expanding the stockpile of AV drugs to encompass the whole UK population (≈60 million) might even be acceptable (≈£6,500 per QALY gained over a no intervention strategy for the 1918 scenario under base-case assumptions).

Stockpiling AVs is a cost-effective option, even though some benefits have been ignored (e.g., possible reduction in CAR if widespread and prompt treatment is offered) (*30*). Furthermore, additional benefits could be derived from using AV drug stockpiles close to their expiration dates to treat epidemic influenza patients. Finally, the reduction in illness and death that may result from widespread AV drug use is likely to bring benefits to more persons and other sectors of the economy.

This study focused on mass treatment strategies because this is the policy of the United Kingdom and other countries (*31*). However, strategies targeting those at higher risk of complications or death would be more cost-effective provided the delivery costs are similar and AV drugs are as effective in these groups.

The main caveat to an AV drug stockpiling strategy is the uncertainty concerning the efficacy of AV drugs against the next strain of pandemic influenza, particularly efficacy against influenza-related deaths. Clearly, estimation of this important parameter should be a priority if governments are to commit large resources to mitigating the effect of an uncertain pandemic occurring at an unknown point in the future.

Use of AV drugs on the scale anticipated may create a selective pressure (any factor that leads to preferential survival of organisms with specific traits) for the emergence of AV drug resistance. In this case, large-scale use of AVs could lead to the preferential survival of flu viruses that are resistant to AVs. However, studies to date indicate that resistance to oseltamivir in influenza virus strains occurs rarely (*32*,*33*) and that such mutations may have a fitness cost in terms of impaired growth and transmissibility (*34*,*35*). Nevertheless, recent reports suggest reduced susceptibility to oseltamivir in some currently circulating strains of avian influenza (H5N1) with pandemic potential (*36*–*38*). Clearly this may reduce the effectiveness of an oseltamivir stockpiling program, although AV drugs might still delay pandemic spread to allow vaccine development (*39*). Further epidemiologic and modeling studies of the potential effect of oseltamivir resistance on viral fitness and drug effectiveness are required.

## Supplementary Material

Appendix Figure Optimal cost-effectiveness of antiviral (AV) and test stockpiling (0–30 million units) for clinical attack rates (CARs) of 15%, 25%, and 35% under the a) 1918 and b) 1957/69 scenarios. The composite test (Test A) and a perfect test of 100% sensitivity and 100% specificity (Test B) are included. The most cost-effective strategies lie on the efficiency line.

Appendix Table Pandemic influenza model parameters
