# Supplementary figures and images for: Cost-effectiveness of Antiviral Stockpiling and Near-Patient Testing for Potential Influenza Pandemic
Source: Emerg Infect Dis. 2008 Feb;14(2):267–74. doi: 10.3201/eid1402.070478 (PMC2600182; doi:10.3201/eid1402.070478)

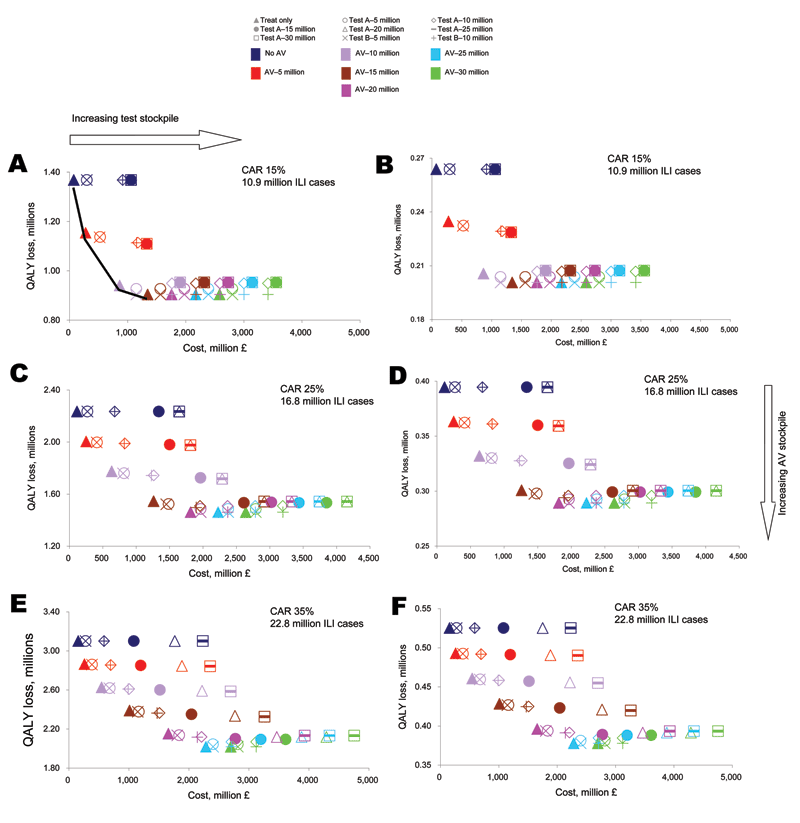

Supplement: Appendix Figure — Optimal cost-effectiveness of antiviral (AV) and test stockpiling (0–30 million units) for clinical attack rates (CARs) of 15%, 25%, and 35% under the a) 1918 and b) 1957/69 scenarios. The composite test (Test A) and a perfect test of 100% sensitivity and 100% specificity (Test B) are included. The most cost-effective strategies lie on the efficiency line. [file 07-0478_app-s1.gif]
