# Supplementary material for: Cost-effectiveness of Antiviral Stockpiling and Near-Patient Testing for Potential Influenza Pandemic
Source: Emerg Infect Dis. 2008 Feb;14(2):267–74. doi: 10.3201/eid1402.070478 (PMC2600182; doi:10.3201/eid1402.070478)
Supplement: Appendix Table — Pandemic influenza model parameters [file 07-0478_appT-s2.pdf]

Appendix Table. Pandemic influenza model parameters

Parameter	Base-case	Distribution	Distribution parameters	Source/assumptions
2004 UK population, millions	59.8	Fixed		(3)
Clinical attack rate	**0.25**	Normal	SD = 0.05	DoH pandemic plan (2)
Pandemic wave, wks	15	Fixed		DoH pandemic plan (2)
Mean weekly ILI consultation rate	**0.00059**	Discrete	p = 0.333	Winter (summer = 0.00018, midwinter = 0.00075) (4)
Proportion ILI patients who consult GP	0.28	Fixed		(5)
Overall CFR (1918 scenario)	0.023	Fixed		HPA unpublished data
Overall CFR (1957/69 scenario)	0.003	Fixed		HPA unpublished data
CFR (1957/69) 0–14 y	0.0001	Fixed		HPA unpublished data
CFR (1957/69) 15–44 y	0.0001	Fixed		HPA unpublished data
CFR (1957/69) 45–64 y	0.0019	Fixed		HPA unpublished data
CFR (1957/69) $\geq$65 y	0.0163	Fixed		HPA unpublished data
Time to pandemic, y	**30**	Discrete uniform	0–59	Assumption
AV drug/test stockpile (millions)	(14.6)	Fixed		Varied in optimal stockpile analysis (0–35) (1)
Wastage	**0.15**	Uniform	0.05–0.25	
Probability of receiving timely AV drugs	**0.70†**	Normal	SD = 0.5	Assumption
Shelf-life of AV drugs, y	5	Triangular	4, 5, 6	(6), assumption
Shelf-life of tests, y	2†	Normal	SD = 0.989	(7–10)
Sensitivity of test	**0.895**	Normal	SD = 0.033	(11–13)
Specificity of test	**0.998‡**	Normal	SD = 0.001	(11–13)
Discount rate for costs and benefits	**0.035**	Fixed		Varied in univariate sensitivity analysis only (0–0.06) (14)
Complications				
OR complication rate, AV drugs vs. none	**0.69**	Normal	SD = 0.085	(15)
Probability GP or A&E consultation (influenza, treated)	**0.05†**	Normal	SD = 0.05	DoH pandemic plan (2), assumption
Probability GP or A&E consultation (noninfluenza ILI or influenza, untreated)	0.068			(15)
OR hospitalization rate, AV drugs vs. none	**0.39§**	Lognormal	Mean = ln (0.39) SD = 0.4103	(15)
Probability of hospitalization				
Influenza, treated	**0.0055**	Normal	SD = 0.001	DoH pandemic plan (2), assumption
Influenza, untreated	0.014			(15)
Noninfluenza ILI	0.006			40% of probability for influenza, untreated (15)

OR death given AV drugs vs. none	**0.39**§	Lognormal	Mean = ln (0.73) SD = 0.4103	Assumed the same as hospitalization
Probability of death (not from influenza)	0.015	Fixed		Mean rate for *S. pneumoniae* (3)
Costs, £				
AV drug per course (treat only)	**16**	Triangular	1, 16, 17	(16), assumption
Near-patient test	**7**	Normal	SD = 2.80	(7–10)
Storage cost of course/test (per y)	**1**	Uniform	1–5	assumption
Administration (per course/test)	15.75†	Normal	SD = £5.09	(17)
Hospitalized (per patient)	**756**	Triangular	± 15%	(17,18), assumption
GP costs (per patient)	**33**	Triangular	± 15%	(17,18), assumption
A&E consultation (per patient)	**81**	Triangular	± 15%	(19), assumption
Health benefits (QALY loss)				
Uncomplicated influenza	0.0046	Normal	SD = 0.0006	0.61 QoL weight, 7 d (20)
Uncomplicated influenza, treated	0.0038¶	Normal	SD = 0.0015	0.65 QoL weight, 7 d (20)
Uncomplicated, noninfluenza	0.0043	Normal	SD = 0.0009	0.62 QoL weight, 7 d (20)
Complications, ILI	0.004	Triangular	± 15%	Pneumonia outpatient (21), assumption.
Hospitalization, ILI#	0.006	Triangular	± 15%	Pneumonia inpatient (21), assumption
QALY loss/influenza death, 1918	16.7			Based on CFR
QALY loss/influenza death, 1957/69	8.1			Based on age-specific CFR
QALY loss/noninfluenza death	6.1			Based on *S. pneumoniae* deaths (3)

*UK, United Kingdom; SD, standard deviation; DoH, Department of Health, ILI, influenza-like illness; CFR, case-fatality ratio; HPA, Health Protection Agency; AV, antiviral; OR, odds ratio; GP, general practitioner; A&E, accident and emergency departments; OR, odds ratio; QALY, quality-adjusted life year; QoL, quality of life. **Boldface** indicates univariate sensitivity analysis.

†Truncated at 0.

‡Truncated at 1.

§Truncated at 0 and 1.

¶Total QALY loss associated with complications is the sum of that associated with pneumonia (outpatient) and the uncomplicated condition.

#Total QALY loss associated with hospitalization is the sum of that associated with pneumonia (inpatient) and the uncomplicated condition.
